# An updated checklist of Azorean arthropods (Arthropoda)

**DOI:** 10.3897/BDJ.10.e97682

**Published:** 2022-12-21

**Authors:** Paulo A. V. Borges, Lucas Lamelas-Lopez, Rui Andrade, Sébastien Lhoumeau, Virgílio Vieira, António Onofre Soares, Isabel Borges, Mário Boieiro, Pedro Cardoso, Luís Carlos Fonseca Crespo, Ole Karsholt, Michael Schülke, Artur Raposo Moniz Serrano, José Alberto Quartau, Volker Assing

**Affiliations:** 1 cE3c- Centre for Ecology, Evolution and Environmental Changes, Azorean Biodiversity Group, CHANGE – Global Change and Sustainability Institute, Faculty of Agricultural Sciences and Environment, University of the Azores, Rua Capitão João d´Ávila, Pico da Urze, 9700-042, Angra do Heroísmo, Azores, Portugal cE3c- Centre for Ecology, Evolution and Environmental Changes, Azorean Biodiversity Group, CHANGE – Global Change and Sustainability Institute, Faculty of Agricultural Sciences and Environment, University of the Azores, Rua Capitão João d´Ávila, Pico da Urze, 9700-042 Angra do Heroísmo, Azores Portugal; 2 IUCN SSC Mid-Atlantic Islands Invertebrates Specialist Group, Angra do Heroísmo, Azores, Portugal IUCN SSC Mid-Atlantic Islands Invertebrates Specialist Group Angra do Heroísmo, Azores Portugal; 3 cE3c- Centre for Ecology, Evolution and Environmental Changes, Azorean Biodiversity Group, CHANGE – Global Change and Sustainability Institute, Faculty of Sciences and Technology, University of the Azores, Rua da Mãe de Deus, 13A, 9500-321, Ponta Delgada, Portugal cE3c- Centre for Ecology, Evolution and Environmental Changes, Azorean Biodiversity Group, CHANGE – Global Change and Sustainability Institute, Faculty of Sciences and Technology, University of the Azores, Rua da Mãe de Deus, 13A, 9500-321 Ponta Delgada Portugal; 4 LIBRe – Laboratory for Integrative Biodiversity Research, Finnish Museum of Natural History Luomus, University of Helsinki, P.O.Box 17 (Pohjoinen Rautatiekatu 13), 00014, Helsinki, Finland LIBRe – Laboratory for Integrative Biodiversity Research, Finnish Museum of Natural History Luomus, University of Helsinki, P.O.Box 17 (Pohjoinen Rautatiekatu 13), 00014 Helsinki Finland; 5 Biodiversity Research Institute UB, Departament of Evolutionary Biology, Ecology and Environmental Sciences (Arthropods), Av. Diagonal 645, E-08028, Barcelona, Spain Biodiversity Research Institute UB, Departament of Evolutionary Biology, Ecology and Environmental Sciences (Arthropods), Av. Diagonal 645, E-08028 Barcelona Spain; 6 Zoological Museum, Natural History Museum of Denmark, Universitetsparken 15, DK–2100, Copenhagen Ø, Denmark Zoological Museum, Natural History Museum of Denmark, Universitetsparken 15, DK–2100 Copenhagen Ø Denmark; 7 Blankenfelder Straße 99, D-13127, Berlin, Germany Blankenfelder Straße 99, D-13127 Berlin Germany; 8 cE3c—Centre for Ecology, Evolution and Environmental Changes, CHANGE – Global Change and Sustainability Institute, Departamento de Biologia Animal, Faculdade de Ciências da Universidade de Lisboa, R. Ernesto de Vasconcelos, Ed. C2, Campo Grande, 1749-016, Lisboa, Portugal cE3c—Centre for Ecology, Evolution and Environmental Changes, CHANGE – Global Change and Sustainability Institute, Departamento de Biologia Animal, Faculdade de Ciências da Universidade de Lisboa, R. Ernesto de Vasconcelos, Ed. C2, Campo Grande, 1749-016 Lisboa Portugal; 9 Gabelsbergerstraße 2, 30163, Hannover, Germany Gabelsbergerstraße 2, 30163 Hannover Germany

**Keywords:** Arthropoda, Azores, diversity patterns, Macaronesia

## Abstract

**Background:**

The Azores is a remote oceanic archipelago of nine islands which belongs to the Macaronesia biogeographical region hosting a unique biodiversity. The present Azorean landscape is strongly modified by the presence of man and only in small areas, where the soil or climate was too rough, have primitive conditions remained unchanged. Despite the fact that most of the Azorean native habitats are now lost, a large number of endemic species are still present and need urgent conservation. The present checklist of terrestrial and freshwater arthropods of the Azores Archipelago is based on all known published literature. The main goal of this work is to list, as rigorously as possible, all the known terrestrial and freshwater arthropods of the Azores. In this way, we are contributing to solve the ‘Linnaean’ shortfall, i.e. an incomplete taxonomic description of species-level diversity and the Wallacean Biodiversity Shortfall, the incomplete species distribution knowledge.

**New information:**

The checklist includes new records of arthropods at island and archipelago levels that were published in the last twelve years. Compared to the last checklist of Azorean arthropods (Borges et al. 2010b), a total of 217 taxa (species and subspecies) are added.

Currently, the total number of terrestrial and freshwater arthropod species and subspecies in the Azores is estimated to be 2420 taxa belonging to 14 classes, 53 orders, 440 families, 1556 genera, 2400 species and 149 individual subspecies.

The most diverse orders of Azorean arthropods are: Coleoptera (585 taxa), Diptera (423 taxa), Hemiptera (338 taxa), Hymenoptera (163 taxa), Lepidoptera (159 taxa) and Araneae (133 taxa).

A total of 276 endemic taxa are currently known (232 species and 44 subspecies), belonging to eight classes and 22 orders. São Miguel, Terceira and Pico are the islands with higher number of endemic species and subspecies. In the Azores, the number of native non-endemic taxa is 793 taxa, totalling 1069 indigenous taxa. Compared to the other nearest Macaronesian archipelagos (Madeira and Canaries), the Azorean arthropod fauna is characterised by a lower percentage of endemism (endemics/indigenous: 26% in Azores, 47% in Madeira Archipelago and 42% in Canary Islands) and a high proportion of exotic introduced taxa (39% in Azores, 19% in Madeira Archipelago and 8% in Canary Islands).

Based on recent IUCN Red-listing of Azorean arthropods, a large fraction of the endemic taxa is under high threat.

## Introduction

**General introduction**

In the last years, novel and more effective methodologies to estimate species richness have been developed (Stork 2018). Global estimations about the number of species of terrestrial arthropods are between 7 and 8.7 million, of which approximately 5.5 million are insects (Ødegaard 2008, Mora et al. 2011, Stork 2018), but an investement in studying morphologically cryptic species will generate higher estimates (Li and Wiens 2022). However, currently only 1.0 million are named, suggesting a large gap between the known and the potential extant diversity (Stork 2018, Li and Wiens 2022). Arthropods are amongst the least represented taxa in global biodiversity datasets, extinction risk assessments and conservation projects (Cardoso et al. 2011, Mammola et al. 2020). Information is lacking on arthropod population sizes, temporal trends, distributions and ecological threats, leaving the conservation status of most arthropod species unknown and unassessed within the context of Red Listing—limiting the implementation of conservation actions (Cardoso et al. 2011). Moreover, the taxonomic impediment that includes the lack of taxonomists and the difficulty in progressing with adequate application of integrative taxonomic approaches to most arthropods, precludes the rapid description of all Earth arthropod diversity (Cardoso et al. 2011, Cardoso et al. 2020, Harvey et al. 2020). The scientific knowledge on taxonomic and functional biodiversity, including their change in space and time, is crucial to support decision-making and planning on many sectors of human activities. These include nature conservation, agriculture, forestry, medical and veterinary sciences. It is important to keep our knowledge updated following regular inventorying and monitoring programmes that contribute with novel findings and provide information on changes in species abundance, richness and composition (Borges et al. 2018c). Additionally to the hyper-diverse insects, arthropods also include spiders, mites, other arachnids, crustaceans, centipedes, millipedes, amongst others and they are the most abundant fauna at global level.

Arthropods play key roles in the ecosystems worldwide (Kremen et al. 1993, Scudder 2009), including the provisioning of an array of fundamental ecosystem services (Ameixa et al. 2018). For example, terrestrial crustaceans, mites, millipedes, springtails and some groups of insects, like true flies and beetles, act as decomposers, by consuming plant litter, dung and corpses, with important implications in nutrient recycling and soil fertility (Culliney 2013, Yang and Gratton 2014). Centipedes, spiders, pseudoscorpions, mites and several groups of insects, like ground- and rove-beetles, ants, wasps, amongst others, are important predators in many ecosystems and they, jointly with many other arthropods, are prey of vertebrate species, thus playing a relevant role in the stability of the trophic webs and ecosystem processes (Staudacher et al. 2018). Additionally, bees, wasps, butterflies, beetles and flies are effective pollinators, directly influencing plant reproduction and indirectly being key to structure ecological communities (Saunders 2017). Finally, it is important to stress that arthropods also provide relevant ecosystem services on human-made ecosystems, as for example, in agroecosystems, through pollination of crops, by enhancing soil fertility and acting as biological control agents. In fact, beetles, ants and spiders are predators of pest insects (Lu et al. 2013, Furlong 2014, Michalko et al. 2019). In addition, granivorous and omnivorous insects, as ground-beetles, can reduce the number of weeds by consuming their seeds (Kulkarni et al. 2017).

Given that arthropods play a key role in all terrestrial ecosystems, they should become a priority for conservation aims. Particularly, the conservation efforts should target insular arthropods communities, since islands host high numbers of endangered endemic species (Kier et al. 2009) that are extremely vulnerable to biotic disturbances, as biological invasions or land-use transformation (Stachowicz and Tilman 2005).

**History of the Azorean arthropod inventories**

Before the foundation of the Azorean Biodiversity Group in 2006, Vieira and Borges (1993) and Borges and Vieira (1994) have compiled the available publications about entomological studies of the Azores (more than 600 publications), being the pioneer publications about the inventorying of Azorean entomofauna.

This century, the number of publications, including arthropod species descriptions and new records to islands, has increased considerably. These publications targeted several terrestrial arthropod groups, but particularly Coleoptera (Soares et al. 2003, Borges et al. 2004, Borges et al. 2007, Borges et al. 2017a, Lamelas-López et al. 2017, Borges et al. 2019, Stüben and Borges 2019, Soares et al. 2021, Borges et al. 2022a), Araneae (Crespo et al. 2013, Crespo et al. 2014, Malumbres-Olarte et al. 2019, Costa and Borges 2021, Carvalho et al. 2021, Crespo et al. 2021), Isoptera (Austin et al. 2012), Oribatida (Subías 2011), Hymenoptera (Weissmann et al. 2017), Hemiptera (Ben-Dov et al. 2012, Lamelas-López et al. 2021) and Lepidoptera (Borges et al. 2018a). Multi-taxa research include also many publications (e.g. Borges et al. 2018b, Arteaga et al. 2020, Borges et al. 2021, Marcelino et al. 2021, Tsafack et al. 2021, Borges et al. 2022b, Tsafack et al. 2022, Lhoumeau et al. 2022a, Lhoumeau et al. 2022b).

## General description

### Purpose

Over one decade after the latest Azorean checklist of terrestrial arthropods (Borges et al. 2010b), we aim to provide an update by including a significant amount of novel information on the Azorean arthropods. In this contribution we: a) perform a detailed taxonomic and nomenclature revision; b) add many new species for the list of Azorean arthropods; c) add many new records for each island.

### Additional information

The present checklist of terrestrial arthropods of the Azores Archipelago is based on all known published literature (see a list of the published literature until 1992 in Vieira and Borges (1993), Borges and Vieira (1994)) and the last checklist of Azorean biota in Borges et al. (2005) and Borges et al. (2010b). A large number of new records for the islands are based on recently published articles (e.g. Stüben and Borges (2019), Carvalho et al. (2021), Costa and Borges (2021), Marcelino et al. (2021), Soares et al. (2021), Tsafack et al. (2021), Borges et al. (2021), Borges et al. (2022a), Borges et al. (2022b), Lhoumeau et al. (2022a), Lhoumeau et al. (2022b)) and one currently in evaluation (e.g. Boieiro et al. (2022)).

For taxonomic consistency, we evaluated specific data for: Araneae, expert revision by PC and LC using the R package arakno (Cardoso and Pekár 2022) that feeds on the World Spider Catalogue (World Spider Catalog 2022); Coleoptera, Staphylinidae (expert revision by VA and MS; see also Borges et al. (2022a)); Coleoptera, Coccinellidae (expert revision by AOS, see also Soares et al. (2021)), Coleoptera, Curculionidae (Stüben and Borges 2019) and Lepidoptera (expert revision by VV and OK).

For the remaining groups, we compared the nomenclature available in five main taxonomic databases: i) the last Azorean Checklist (Borges et al. 2010b); ii) the project Fauna Europaea (https://fauna-eu.org/); iii) the project BIOTA Canarias (https://www.biodiversidadcanarias.es/), iv) the GBIF - Global Biodiversity Information Facility database (https://www.gbif.org/), when data were available; and v) the project Fauna Iberica (http://www.fauna-iberica.mncn.csic.es/english/). In general, we looked for consistency in the nomenclature and when in doubt, we followed the GBIF nomenclature.

We also made a consultation of GBIF for recent additions and discovered several new species added for Azores, mostly from Museum records and iNaturalist. For now, we do not add these records to the current checklist, but provide the data as a Supplement to this manuscript (Suppl. material 1). When these records are confirmed by our team, they will be added in future editions of our Checklist.

In analytical tables, both in main body of the manuscript and in Supplementary Tables, higher taxa are listed phylogenetically, in a sequence inferred to be from the less to the more derived groups, with closely-related taxa placed near to one another. The families, genera and species names are listed in alphabetical sequence. Synonyms include true synonyms, names resulting from misidentifications and typographical errors. Synonyms can be seen in AZORESBIOPORTAL (https://azoresbioportal.uac.pt/) associated with each species webpage.

Information on the distribution of species and subspecies at island level in the Azores Archipelago is presented using the following abbreviations: **COR** - Corvo Island; **FLO** - Flores Island; **FAI** - Faial Island; **PIC** - Pico Island; **GRA** - Graciosa Island; **SJG** - São Jorge Island; **TER** - Terceira Island; **SMG** - São Miguel Island; and **SMR** - Santa Maria Island.

When no information concerning island occurrence was available, only archipelago occurrence is given (AZ). In most cases, this corresponds to old records, as well as to references to the Azores, as found in “Fauna Europaea”, with no indication to any literature supporting these findings.

Abbreviations of the colonisation status of each species presented in the checklist are as follows:

- **END – Azorean endemics**, i.e. species or subspecies occurring only in the Azores, as a result of either speciation events (neo-endemics) or extinction of the mainland populations (palaeo-endemics);
- **MAC – Macaronesian endemic species**, i.e. species only known from Macaronesia (Azores, Madeira, Salvages, Canaries and Cabo Verde Islands);
- **NAT – native non-endemic species**, i.e. species which arrived by long-distance dispersal in the Azores and which also occur in other archipelagos and/or on continents;
- **M – migrant species**, i.e. butterflies, moths and dragonflies which arrived to the Azores by long-distance dispersal, such as migratory flights, periodically (seasonal migrants) or occasionally (non-seasonal migrants); some of them establish breeding populations (e.g. Monarch butterfly);
- **INTR – introduced species**, i.e. species believed to occur in the archipelago as a result of human activities; some of these species have a worldwide distribution;
- **INDT – indeterminate**, i.e. species for which no information is available to decide on the correct colonisation status.

The native and introduced status of a taxon is only given for the taxa of which there is published information or by taking into consideration the expertise of the taxonomic coordinator.

## Project description

### Title

The Checklist of Azorean Arthropods

### Personnel

Paulo A.V. Borges conceived and coordinated the project.

Many taxonomists contributed with information and shared fieldwork during the last thirty years. The details on these contributions can be seen in the two previous Checklists of Azorean Arthropods (Borges et al. 2005, Borges et al. 2010b).

### Study area description

**Figure 1.**
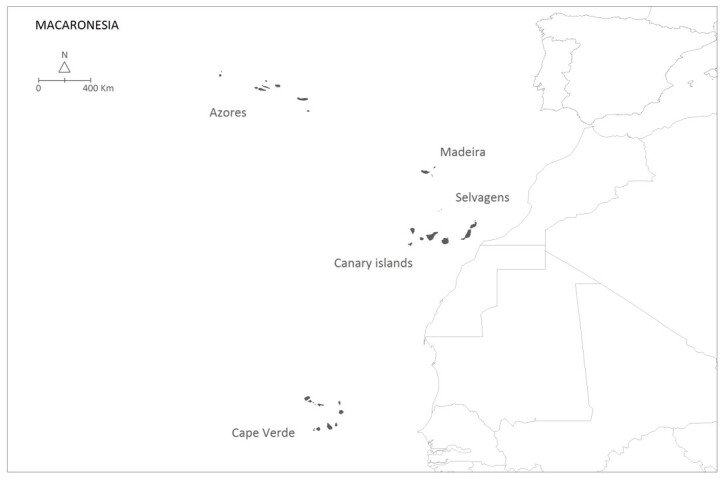
The Azores Archipelago location in mid-Atlantic.

**Figure 2.**
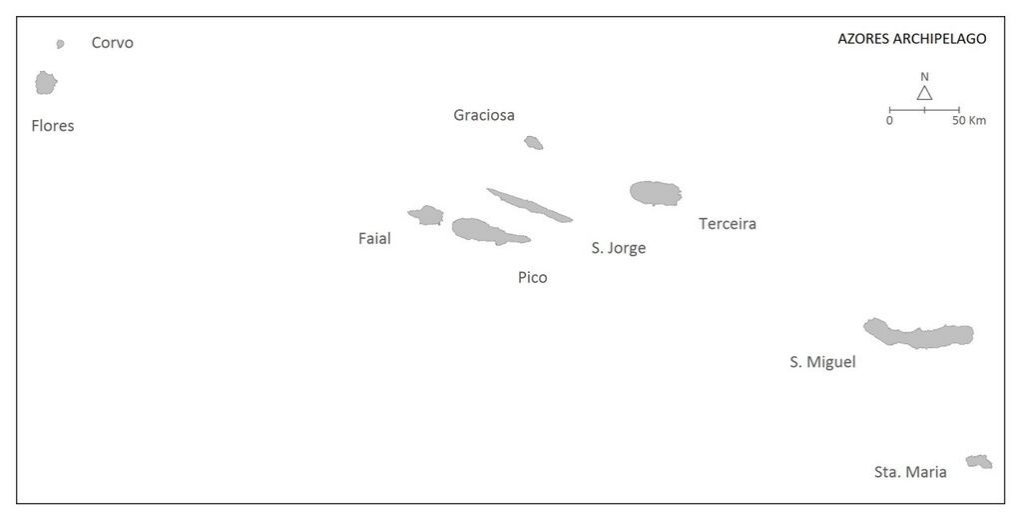
The nine Azorean Islands.

The Azores are an isolated archipelago (38°43′49″N, 27°19′10″W, Fig. 1), situated in the mid-Atlantic Ocean comprising nine volcanic Islands spread over 500 km in a W/NW–E/SE direction (Fig. 2).

### Funding

This work was financed by three main projects:

- (2016-2022) - Fundação da Ciência e Tecnologia - FCT-UIDB/00329/2020-2024.
- (2019-2022) - FEDER - AZORESBIOPORTAL –PORBIOTA (ACORES-01-0145-FEDER-000072).
- (2022-2023) - Portal da Biodiversidade dos Açores - PO Azores Project - M1.1.A/INFRAEST CIENT/001/2022.

## Geographic coverage

### Description

Azores Islands: COR - Corvo Island; FLO - Flores Island; FAI - Faial Island; PIC - Pico Island; GRA - Graciosa Island; SJG - São Jorge Island; TER - Terceira Island; SMG - São Miguel Island and SMR - Santa Maria Island.

### Coordinates

36.77409249464195 and 39.9602803542957 Latitude; -31.39892578125 and -24.85107421875 Longitude.

## Taxonomic coverage

### Description

All terrestrial and freshwater Arthropoda.

## Usage licence

### Usage licence

Creative Commons Public Domain Waiver (CC-Zero)

## Data resources

### Data package title

Updated Checklist of Arthropods from Azores (Portugal)

### Resource link

https://www.gbif.org/dataset/2d91cfd8-0a48-4d80-8128-080e52a1e650

### Alternative identifiers

http://ipt.gbif.pt/ipt/resource?r=checklist_arthropoda_azores&v=1.5

### Number of data sets

2

### Data set 1.

#### Data set name

Taxon Table

#### Data format

Darwin Core Archive

#### Character set

UTF-8

#### Download URL

http://ipt.gbif.pt/ipt/resource?r=checklist_arthropoda_azores&v=1.5

#### Data format version

version 1.6

#### Description

The complete list of species and subspecies with the distribution per island is available in Suppl. material 2. In this supplementary material, there is a code (ABPCODE) associated to each taxon that connects to the AZORESBIOPORTAL. The AZORESBIOPORTAL (Borges et al. 2010a) is an online database on the Azores biodiversity that is being updated continuously since 2006 with novel information on the taxonomy, ecology and distribution of terrestrial arthropods. The current version of this website (see https://azoresbioportal.uac.pt/) now lists about 11,483 species of all types of organisms, most of them (68%) terrestrial.

**Taxon Dataset in GBIF**: The dataset was published in GBIF - Portugal (Borges et al. 2022c). The published data include all the taxa listed in Suppl. material 2. The dataset submitted to GBIF is structured as a Checklist dataset that has been published as a Darwin Core Archive (DwCA), which is a standardised format for sharing biodiversity data as a set of one or more data tables. The Taxon Table contains 4614 records. This GBIF IPT (Integrated Publishing Toolkit, Version 2.6.2) archives the data and, thus, serves as the data repository. We provide this Darwin Core Archive (DwCA) in Supplementary Material for taxon data (Suppl. material 3).

**Data set 1.** 

Column label	Column description
id	Identifier of the record, unique for the dataset.
taxonID	Identifier of the taxon, unique for the dataset.
acceptedNameUsageID	Identifier for the name usage of the currently valid taxon.
parentNameUsageID	Identifier for the name usage of the direct, most proximate higher-rank parent taxon of the most specific element of the scientificName.
taxonRemarks	The ABPCODE that connects each species or subspecies to AZORESBIOPORTAL.
scientificName	Complete scientific name including author and year.
acceptedNameUsage	Complete scientific name including author and year, of the currently valid taxon.
parentNameUsage	Complete scientific name including author and year, of the direct, most proximate higher-rank parent taxon of the most specific element of the scientificName.
kingdom	Kingdom name.
phylum	Phylum name.
class	Class name.
order	Order name.
family	Family name.
genus	Genus name.
specificEpithet	Specific epithet.
infraspecificEpithet	Infrapecific epithet.
taxonRank	Lowest taxonomic rank of the record.
taxonomicStatus	The status of the use of the scientificName as a label for a taxon.
scientificNameAuthorship	Name of the author of the lowest taxon rank included in the record.
modified	The most recent date-time on which the resource was changed.
nomenclaturalCode	The nomenclatural code under which the scientificName is constructed (ICNZ in the current case).
language	The language of the resource.
licence	Legal information giving official permission to do something with the resource.
rightsHolder	Institution owning or managing rights over the resource.
datasetID	Identifier of the dataset.
institutionID	Identifier of the institution.
institutionCode	Name of the institution.
datasetName	Name of the dataset.

### Data set 2.

#### Data set name

Distribution Table

#### Data format

Darwin Core Archive

#### Character set

UTF-8

#### Download URL

http://ipt.gbif.pt/ipt/resource?r=checklist_arthropoda_azores&v=1.5

#### Data format version

version 1.6

#### Description

The complete list of species and subspecies with the distribution per island is available in Suppl. material 2. In this supplementary material, there is a code (ABPCODE) associated with each taxon that connects to the AZORESBIOPORTAL.

**Distribution Dataset in GBIF**: The dataset was published in the Global Biodiversity Information Facility platform, GBIF (Borges et al. 2022c). The published data include all the taxa listed in Suppl. material 2. The dataset submitted to GBIF is structured as a Checklist dataset that has been published as a Darwin Core Archive (DwCA), which is a standardised format for sharing biodiversity data as a set of one or more data tables. The Distribution Table contains 8630 records about detailed distribution data. This GBIF IPT (Integrated Publishing Toolkit, Version 2.6.2) archives the data and, thus, serves as the data repository. We provide this Darwin Core Archive (DwCA) in Supplementary Material for distribution data (Suppl. material 4).

**Data set 2.** 

Column label	Column description
taxonID	Identifier of the taxon, unique for the dataset.
identificationRemarks	The ABPCODE that connects each species or subspecies to AZORESBIOPORTAL.
locality	Name of the locality.
locationID	Identifier of the location.
establishmentMeans	The process of establishment of the species in the location, using a controlled vocabulary: in the GBIF database, we used the notation: 'endemic', 'native', "Macaronesia", 'introduced', "migrant", "indeterminate".
occurrenceRemarks	Additional information on the occurrence of the species.

## Additional information

**Checklist of Azorean terrestrial and freshwater arthropods**

### Analysis and Discussion

***Species richness patterns***

**Figure 3.**
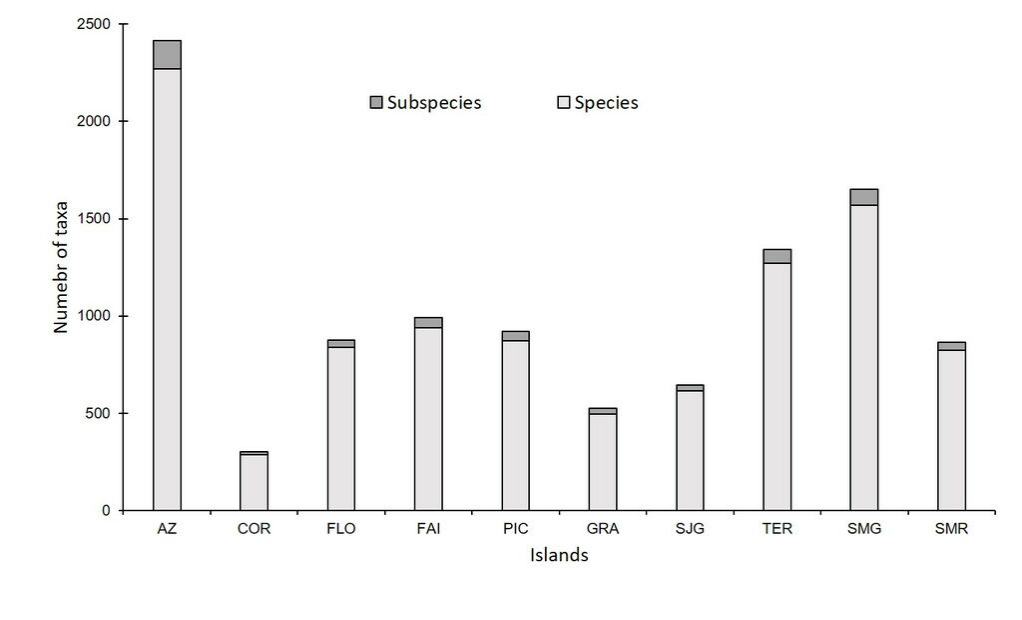
Number of taxa (species and subspecies) of terrestrial arthropods in the Azores Archipelago (AZ), for the islands of Corvo (COR), Flores (FLO), Faial (FAI), Pico (PIC), Graciosa (GRA), São Jorge (SJG), Terceira (TER), São Miguel (SMG) and Santa Maria (SMR).

A total of 2420 taxa of terrestrial and freshwater arthropods are listed for the Azores, belonging to 14 classes and 53 orders (Fig. 3; Suppl. material 5). The most species-rich islands are São Miguel and Terceira, due to their larger sizes and historically more intensive sampling effort. Despite being the second in size, Pico Island comes only as fourth in number of taxa, mostly due to its recent geological age (Borges and Brown 2008). Corvo, Graciosa and São Jorge Islands are the least diverse, probably due to their smaller size and lower sampling frequency. Remarkably, Santa Maria and Faial are relatively biodiverse islands in relation to their sizes (Fig. 3). The diversity patterns in relation to total number of taxa (species + subspecies) are relatively similar across the islands (Fig. 3).

In comparison with other Macaronesian archipelagos, Azores has the lowest arthropod diversity, a consequence of the combination of several factors, namely: i) recent geological age (a larger fraction of the terrain has less than 1 My); ii) very homogeneous landscape with low habitat diversity; and iii) geographical isolation (see Triantis et al. (2012)). For example, Madeira and Selvagens Archipelagos host around 3900 arthropod taxa (Borges et al. 2008), which can be explained by the fact that these islands are nearer to the mainland, their geological history is older and, concerning Madeira Island, its complex topography and habitat diversity are comparatively higher than any Azorean island. Azores has also a high proportion of exotic introduced taxa (39% in Azores, 19% in Madeira Archipelago and 8% in Canary Islands).

**Figure 4.**
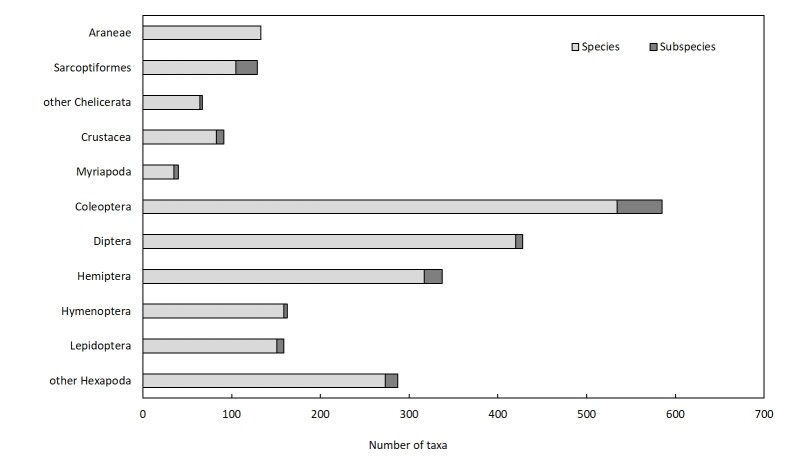
Number of species and subspecies of the most diverse terrestrial arthropods groups.

The most diverse orders of Azorean terrestrial and freshwater arthropods belong to the Insecta and Arachnida (Fig. 4): Coleoptera (585 taxa), Diptera (422 taxa), Hemiptera (338 taxa), Hymenoptera (162 taxa), Lepidoptera (159 taxa) and Araneae (132 taxa).

***Endemism patterns***

Oceanic islands frequently harbour lower number of species per unit area in comparison with the mainland, but the proportion of endemic taxa is, in general, higher in the former (Whittaker and Fernández-Palacios 2007, Kier et al. 2009). Therefore, oceanic islands are considered hotspots of biodiversity at global level (Kier et al. 2009).

**Figure 5.**
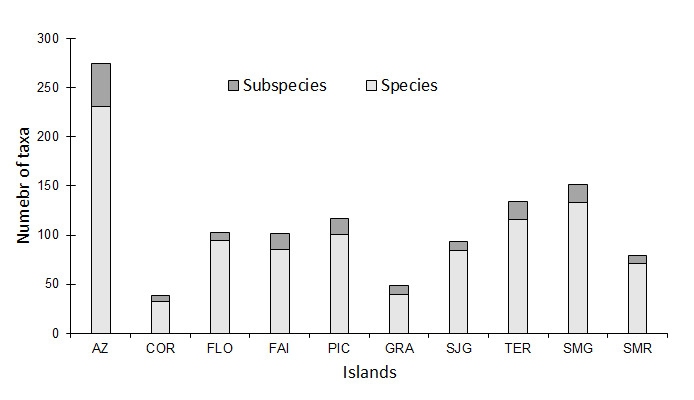
Number of endemic species of terrestrial arthropods in the Azores Archipelago (AZ), for the islands of Corvo (COR), Flores (FLO), Faial (FAI), Pico (PIC), Graciosa (GRA), São Jorge (SJG), Terceira (TER), São Miguel (SMG) and Santa Maria (SMR).

In the case of the Azores, a total of 276 endemic taxa (232 species and 44 subspecies) occur in the Archipelago (Fig. 5; Suppl. material 6), belonging to eight classes and 22 orders. São Miguel, Terceira and Pico are the islands with higher number of endemic species and subspecies. Corvo, Flores and Santa Maria are the islands with lower number of endemic species and subspecies.

Concerning the number and proportion of Single Island Endemics (SIE), i.e. the endemic species restricted to a single island, from a total of 122 SIEs, São Miguel and Santa Maria host the larger proportion in relation to the total endemics per island, which is explained by their older geological ages (Borges and Brown 2008, Triantis et al. 2012).

In terms of endemic species, Azores Archipelago also harbours lower number of species in comparison with the other Macaronesian archipelagos (276 endemic taxa; Borges et al. 2010b). In contrast, Madeira and Selvagens Archipelagos host around 1000 (Borges et al. 2008) and the Canary Islands around 2900 endemic taxa (Arechavaleta et al. 2009). In the Azores, the number of native non-endemic taxa is 793 taxa, totalling 1069 indigenous taxa. Compared to the other nearest Macaronesian archipelagos (Madeira and Canaries), the Azorean arthropod fauna is characterised by a lower percentage of endemism (endemics/indigenous: 26% in Azores, 47% in Madeira Archipelago and 42% in Canary Islands).

**Figure 6.**
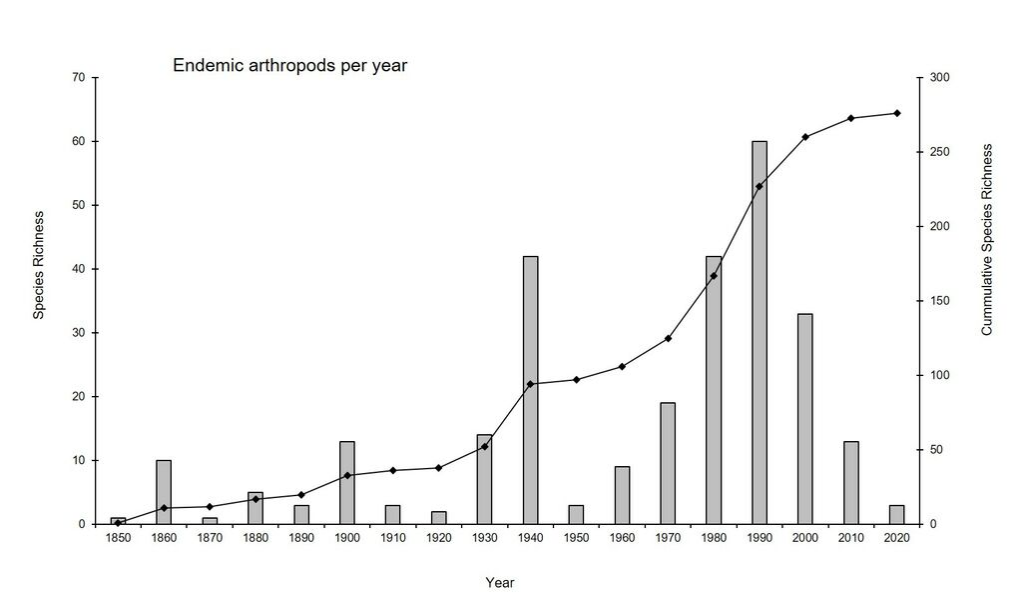
Cumulative discovery curve of the Azores Archipelago endemic arthropod species and subspecies. Bars depict the number of endemic taxa described in each decade and the curve their accumulation over time.

The most diverse orders, in terms of endemic species and subspecies, were Coleoptera (80 taxa), Diptera (48 taxa), Lepidoptera (40 taxa), Sarcoptiformes (27 taxa) and Araneae (26 taxa) (Suppl. material 6). The rate at which the records of new species have occurred over the last few years, translated in the cumulative discovery curve (Fig. 6), highlights the great efforts to study Azorean arthropods since 1850. Since the 40s' decade and particularly from 1980, the discovery rate increased considerably, from 52 endemic species reported in 1930 (18% of the total), to 230 before 2000 (around 82%; Fig. 6). In the last 20 years, several publications described numerous new species, including Coleoptera (Borges et al. 2004, Borges et al. 2007, Borges et al. 2017a) and Araneae (Crespo et al. 2013, Crespo et al. 2014, Crespo et al. 2021). The publications on the inventorying and monitoring of Azorean terrestrial arthropods have been increased since the consolidation of the Azorean Biodiversity Group and the University of the Azores. Therefore, given that the cumulative curve of endemic taxa discovery seems to be reaching the asymptote, this suggest that the inventory of Azorean endemic arthropods is, at least for the most studied taxa, almost complete. However, new infra-sampled or artificial microhabitats (e.g. the role of artificial water reservoirs to macroinvertebrates, Lamelas-López et al. (2021)) can host new endemic species, as occurred in the case of volcanic caves (e.g. Borges et al. (2004)). Moreover, poorly-studied groups like Acari, Diptera, Micro-Lepidoptera and Hymenoptera need further taxonomic effort and Lobo and Borges (2010) suggest that indeed the inventory of endemic species is far from complete.

Finally, some arthropod genera contain a high number of endemic taxa (four or more species and subspecies). Coleoptera genera with higher number of endemic taxa include *Tarphius* (12 taxa), *Drouetius* (nine taxa), *Trechus* (nine taxa) and *Calathus* (four taxa) (Borges et al. 2017a, Borges et al. 2019); Diptera includes the genus *Kowarzia* (four taxa); Hemiptera includes the genus *Cixius* (11 taxa); and Lepidoptera include the genera *Hipparchia* (three taxa), *Phlogophora* (four taxa) and *Scoparia* (four taxa) (Borges et al. 2018a).

***IUCN Red List Assessments***

The International Union for Conservation of Nature (IUCN) Red List provides data to inform on the health of the world’s biodiversity. It is a powerful tool to provide information and catalyse action for biodiversity conservation and policy change, critical to protecting natural resources. The IUCN Red List for threatened species identifies the conservation status of species including their extinction risk and simultaneously provides information on the health of local to global biodiversity. The IUCN assessments attribute a Red List category to a species following the application of specific criteria and provide information about its range, population sizes, description of habitat and ecology, main threats and conservation actions needed. The use of this valuable information may allow the implementation of more precise, efficient and effective conservation actions and drive policy changes, focused to protect the natural resources and biodiversity.

In the Azores, a total of 260 species were assessed during the last decade (see http://www.maiisg.com), mainly in the last five years. A summary of the IUCN Red List profiles are available for 54 Coleoptera (Borges et al. 2017b), 15 cave-adapted arthropods (Borges et al. 2019) and 34 Lepidoptera (Borges et al. 2018a) endemic species.

**Figure 7.**
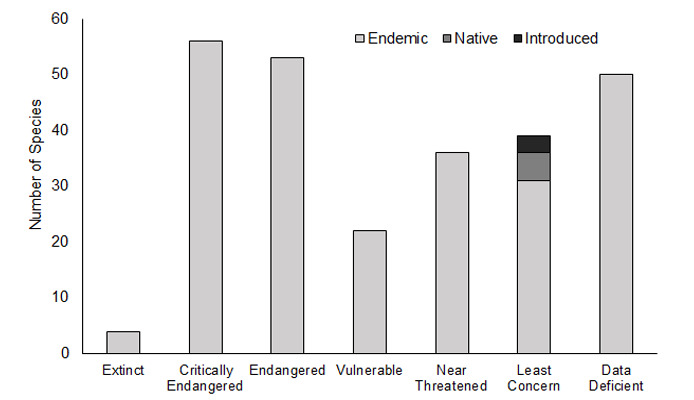
IUCN Red List Assessments categories for endemic, native and introduced terrestrial arthropod species in the Azores.

Most of the assessed species are endemic; however, assessments were also performed for five native and three introduced species, although their category is “Least Concern” (Fig. 7). Most of the endemic species are threatened in the Azores probably as a result of the spread of invasive plant species, habitat loss associated with landscape transformation or due to climate change effects (Triantis et al. 2012, Ferreira et al. 2016, Borges et al. 2020). In particular, four endemic species are extinct in Azores and 56, 53 and 22 are critically endangered, endangered or vulnerable, respectively (Fig. 7). Additionally, a total of 36 species are classified as Near Threatened, raising great concern on their future population trends, if specific conservation actions are not implemented in the next few years. Additionally, a considerable number of endemic species were classified as Data Deficient, pinpointing the need to address resources to collect basic data on their abundance, distribution and ecology.

**Figure 8.**
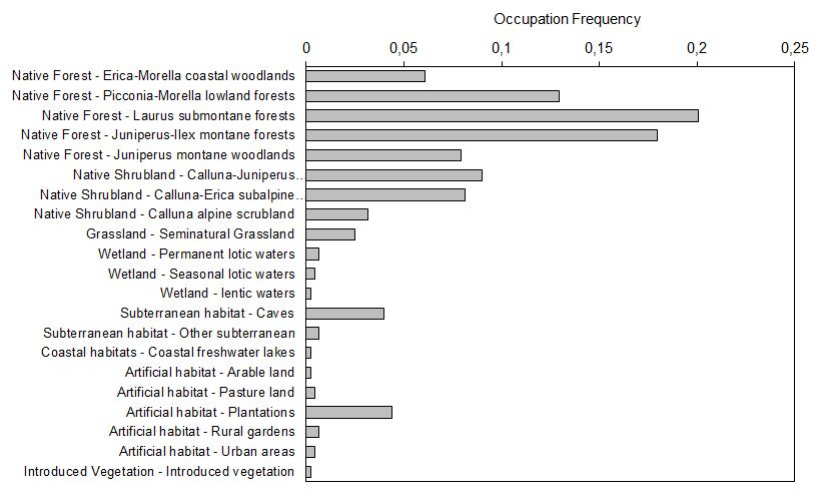
Main habitats occupied by the endemic species, according to IUCN Red List criteria. Bars represent the occupation frequency of a habitat by the endemic species, given that some species occur in several habitats.

Native forests are the most important habitats for endemic arthropods of the Azores (around 64% of species depend on them: Fig. 8). These forests have high conservation value, being dominated by *Ericaazorica*, *Morellafaya*, *Picconiaazorica*, *Laurusazorica*, *Ilexazorica* and *Juniperusbrevifolia*. Pristine native forests harbour unique Coleoptera endemic species, some of them possibly already extinct (Terzopoulou et al. 2015) or under severe threats (Triantis et al. 2010, Ferreira et al. 2016). Other important native habitats include native shrublands, caves and other subterranean habitats and semi-natural grasslands.

Invasive plant species and habitat transformation by exotic tree plantations are some of main threats to native landscapes and endemic species in the archipelago (e.g. Triantis et al. (2010), Borges et al. (2020)). However, it is remarkable that even human-made habitats can also host populations of native and endemic species. For example, a recent study found that lowland patches of exotic forests, close to native forests or included in a matrix of other artificial areas, such as agro-ecosystems, can sustain populations of rare endemic species, playing an important role in the conservation of the Azorean native arthropods (Tsafack et al. 2021).

### Temporal patterns in species additions

Borges et al. (2005) created the first checklist of Azorean arthropods, reporting a total of 2187 species, including information of presence at island level and about colonisation status (endemic, introduced or native species). Five years later, an updated checklist was published (Borges et al. 2010b), increasing the number of Azorean terrestrial arthropod species up to 2203. Currenty this number of species and subspecies has increased to 2420. In the Table 1 and Table 2, we present the number of records of total and endemic taxa recorded in the aforementioned publications.

***Future perspectives***

Currently, we are conducting a long-term monitoring of Azorean arthopods in native forests (see Lhoumeau et al. (2022a), Lhoumeau et al. (2022b)) which are contributing with many new records for the Azores Islands, but more importantly, providing information about the trends in species temporal and spatial turnover (Matthews et al. 2019, Borges et al. 2020). The Azores constitute an ideal model system for a long-term monitoring study because: 1) they possess a unique forest type in Europe, resembling the lost temperate forests of the Tertiary and about 5% of which remain, including some pristine areas of great ecological importance (Triantis et al. 2010); 2) they are one of the most isolated archipelagos in the world, harbouring a significant number of single island endemics.

The preservation of the unique biodiversity of the Azores Archipelago is critical and, with this new list of the Azorean arthropod biodiversity, we hope to provide a stimulating context for the learning about biodiversity and to foster collaboration amongst taxonomists and ecologists interested in island ecosystems. In addition, we believe that this work will contribute to support further research and conservation actions aiming to preserve the diversity of the Azores and hope that it will also help all those needing details on the taxonomy and nomenclature of the Azorean arthropods.

## Supplementary Material

D0E6CDB4-135A-5EDE-83CC-B24DE70DFA3C 10.3897/BDJ.10.e97682.suppl1 Supplementary material 1 List of additional species (by GBIF) pending confirmation Data type Occurrences in GBIF Brief description Detailed list of of species recorded in GBIF mostly from Natural History Museums and iNaturalist. In addition to the taxonomy details, we include information on type of record (Preserved specimen, Human observation), the name of the recorder person, the link for the GBIF identifier as occurrenceID, the person that identified the species and date, the island of occurrence and the dataset. File: oo_770074.txt https://binary.pensoft.net/file/770074 Paulo A. V. Borges, Sébastien Lhoumeau & Rui Andrade

CED25A24-C7BB-58F9-B863-28A748BA8F40 10.3897/BDJ.10.e97682.suppl2 Supplementary material 2 Complete list of Azorean Arthropods Data type Occurrences Brief description Information on the distribution of species and subspecies at island level in the Azores Archipelago is presented using the following abbreviations: ABPCODE: Code of the taxon in AZORESBIOPORTAL; COR - Corvo Island; FLO - Flores Island; FAI - Faial Island; PIC - Pico Island; GRA - Graciosa Island; SJG - São Jorge Island; TER - Terceira Island; SMG - São Miguel Island; and SMR - Santa Maria Island. When no information concerning island occurrence was available, only archipelago occurrence is given (AZ). In most cases, this corresponds to old records, as well as to references to the Azores, as found in “Fauna Europaea”, with no connection to any literature supporting these findings. Abbreviations of the colonisation status of each species presented in the checklist are as follows: END – Azorean endemics, i.e. species or subspecies occurring only in the Azores, as a result of either speciation events (neo-endemics) or extinction of the mainland populations (palaeo-endemics); MAC – Macaronesian endemic species, i.e. species only known from Macaronesia (Azores, Madeira, Canaries and Cabo Verde Islands); NAT – native non-endemic species, i.e. species which arrived by long-distance dispersal in the Azores and which also occur in other archipelagos and/or on continents; M – migrant species, i.e. butterflies, moths and dragonflies which arrived to the Azores by long-distance dispersal, such as migratory flights, periodically (seasonal migrants) or occasionally (non-seasonal migrants); some of them establish breeding populations (e.g. Monarch butterfly); INTR – introduced species, i.e. species believed to occur in the Archipelago as a result of human activities; some of these species have a worldwide distribution; INDT – indeterminate, i.e. species for which no information is available to decide on the correct colonisation status. File: oo_782207.txt https://binary.pensoft.net/file/782207 Paulo A. V. Borges, Lucas Lamelas-Lopez, Rui Andrade, Sébastien Lhoumeau, Virgílio Vieira, António O. Soares, Isabel Borges, Mário Boieiro, Pedro Cardoso, Luís Carlos Crespo, Ole Karsholt, Volker Assing, Michael Schülke, Artur R.M. Serrano & José Alberto Quartau

BE09C614-19A8-505B-9B56-EFA725DDBFE7 10.3897/BDJ.10.e97682.suppl3 Supplementary material 3 Darwin Core database - Updated Checklist of Azorean Arthropods (Taxon) Data type Taxonomic List Brief description GBIF Darwin Core Dataset - Taxon data. File: oo_782208.txt https://binary.pensoft.net/file/782208 Borges, P.A.V., Lamelas-Lopez, L., Andrade, R., Lhoumeau, S., Vieira, V., Soares, A.O., Borges, I., Boieiro, M., Cardoso, P., Crespo, L.C., Karsholt, O., Assing, V., Schülke, M., Serrano, A.R.M. & Quartau, J.A.

7C56DD3D-2501-5C2F-90AA-256F7141B714 10.3897/BDJ.10.e97682.suppl4 Supplementary material 4 Darwin Core database - Updated Checklist of Azorean Arthropods (Distribution) Data type Distribution data Brief description GBIF Darwin Core Dataset - Distribution data. File: oo_782210.txt https://binary.pensoft.net/file/782210 Borges, P.A.V., Lamelas-Lopez, L., Andrade, R., Lhoumeau, S., Vieira, V., Soares, A.O., Borges, I., Boieiro, M., Cardoso, P., Crespo, L.C., Karsholt, O., Assing, V., Schülke, M., Serrano, A.R.M. & Quartau, J.A.

014ABB76-A2C4-5639-B910-AEC9327B7F78 10.3897/BDJ.10.e97682.suppl5 Supplementary material 5 Table of total taxa (species and subspecies) recorded in the updated Azorean arthropods checklist. Data type Occurrences data per island Brief description Table compiling the total taxa (species and subspecies) of terrestrial arthropods in the Azores Archipelago (AZ), for the islands of Corvo (COR), Flores (FLO), Faial (FAI), Pico (PIC), Graciosa (GRA), São Jorge (SJG), Terceira (TER), São Miguel (SMG) and Santa Maria (SMR). File: oo_782211.txt https://binary.pensoft.net/file/782211 Paulo A.V. Borges, Lucas Lamelas López.

C2ABB050-CB15-594D-8FB3-2AAC00032657 10.3897/BDJ.10.e97682.suppl6 Supplementary material 6 Total of endemic taxa (species and subspecies) recorded in the updated Azorean arthropods checklist. Data type Occurrences data per island Brief description Table compiling the endemic taxa (species and subspecies) of terrestrial arthropods in the Azores Archipelago (AZ), for the islands of Corvo (COR), Flores (FLO), Faial (FAI), Pico (PIC), Graciosa (GRA), São Jorge (SJG), Terceira (TER), São Miguel (SMG) and Santa Maria (SMR). File: oo_782214.txt https://binary.pensoft.net/file/782214 Paulo A.V. Borges, Lucas Lamelas-López

## Figures and Tables

**Table 1.** Number of recorded total taxa (species and subspecies) in the Azorean checklists of 2005 (Borges et al. 2005), 2010 (Borges et al. 2010b) and the current one.

**Taxonomic group**			**Mumber of taxa (species and subspecies)**		
Subphylum	Class	Order	2005	2010	2022
Chelicerata	Arachnida	Araneae	121	124	133
		Ixodida	11	10	11
		Mesostigmata	24	23	24
		Opiliones	2	3	3
		Pseudoscorpiones	8	10	10
		Sarcoptiformes	113	110	129
		Trombidiformes	17	19	19
Crustacea	Branchiopoda	Anomopoda	9	9	9
		Ctenopoda	1	1	1
		Haplopoda	1	1	1
	Hexanauplia	Calanoida	3	3	3
		Cyclopoida	11	11	11
		Harpacticoida	5	5	5
	Ichthyostraca	Arguloida	1	1	1
	Malacostraca	Amphipoda	14	13	13
		Decapoda	1	1	1
		Isopoda	39	32	32
	Ostracoda	Podocopida	14	14	14
Myriapoda	Chilopoda	Geophilomorpha	6	6	6
		Lithobiomorpha	6	6	6
		Scolopendromorpha	1	1	1
		Scutigeromorpha	1	1	1
	Diplopoda	Chordeumatida	1	1	1
		Julida	12	12	12
		Polydesmida	8	8	8
		Polyxenida	1	1	1
	Pauropoda	Tetramerocerata	1	1	1
	Symphyla	Symphyla	3	3	3
Hexapoda	Collembola	Entomobryomorpha	43	45	48
		Neelipleona	3	3	3
		Poduromorpha	26	26	26
		Symphypleona	21	21	24
	Diplura	Diplura	3	3	3
	Protura	Protura	2	2	2
	Insecta	Archaeognatha	4	4	4
		Blattodea	7	6	11
		Coleoptera	528	531	585
		Dermaptera	5	5	5
		Diptera	393	406	428
		Ephemeroptera	1	1	1
		Hemiptera	304	306	338
		Hymenoptera	131	128	163
		Lepidoptera	149	151	159
		Neuroptera	7	7	7
		Odonata	4	4	7
		Orthoptera	14	15	16
		Phasmida	1	2	2
		Psocodea	36	36	55
		Siphonaptera	15	15	15
		Strepsiptera	1	1	1
		Thysanoptera	47	49	49
		Trichoptera	4	3	5
		Zygentoma	3	3	3
					
TOTAL			2187	2203	2420

**Table 2.** Number of recorded endemic taxa (species and subspecies) in the Azorean checklists of 2005 (Borges et al. 2005), 2010 (Borges et al. 2010b) and the current one.

**Taxonomic group**			**Number of endemic taxa (species and subspecies)**		
Subphylum	Class	Order	2005	2010	2022
Chelicerata	Arachnida	Araneae	23	22	26
		Pseudoscorpiones	3	2	2
		Sarcoptiformes	27	27	27
Crustacea	Hexanauplia	Cyclopoida	2	2	2
	Malacostraca	Amphipoda	4	4	4
		Isopoda	7	2	2
Myriapoda	Chilopoda	Lithobiomorpha	3	3	3
	Diplopoda	Polydesmida	1	1	1
	Pauropoda	Tetramerocerata	1	1	1
Hexapoda	Collembola	Entomobryomorpha	2	2	2
		Poduromorpha	1	1	1
	Insecta	Archaeognatha	2	2	2
		Coleoptera	66	72	80
		Diptera	52	48	48
		Hemiptera	18	19	18
		Hymenoptera	11	9	11
		Lepidoptera	38	37	40
		Neuroptera	1	1	1
		Orthoptera	1	1	1
		Psocodea	2	2	2
		Thysanoptera	1	1	1
		Trichoptera	1	1	1
		TOTAL	267	260	276
